# 3rd National Conference Academic Medical Centre: Health System Resilience During the Covid-19 Pandemic: Virtual. 22-23 March 2021

**DOI:** 10.1186/s12919-021-00222-7

**Published:** 2021-08-26

## Keynotes

### KN1 Setting the Scene: An Overview of the COVID-19 Pandemic in Malaysia

#### Rohaizat Yon (drrohaizat.yon@gmail.com)

##### Former-Deputy Director-General (Medical), Ministry of Health, 62200 Putrajaya, Malaysia

**Abstract**

At the beginning of the pandemic, only six Ministry of Health (MOH) laboratories were responsible for conducting 832 COVID-19 tests per day. Currently, COVID-19 patients and patients under investigation have access to 466 ICU beds, with a 42 per cent utilization rate. After the outbreak of the COVID-19 pandemic, the MOH has established COVID-19 care guidelines that are revised regularly and consistent with recommendations from international organizations such as the World Health Organization and scientific studies and treatment effectiveness for patients in Malaysia and abroad. Since we lack specialization in treating COVID-19, patients are treated based on the classification of COVID-19 infection, clinical condition, laboratory and imaging test results. In Malaysia, all patients who are confirmed positive for COVID-19, including those who are asymptomatic, will be treated at the designated treatment centres, either in the hospital or Quarantine and Low-Risk COVID-19 Treatment Centres (PKRC). As a result, COVID-19 patients are isolated from the community. This approach is effective in reducing the risk of COVID-19 infections from spreading wider into the community. The treatment centres will closely monitor the clinical progress of COVID-19 patients in terms of vital signs, blood tests, and imaging according to patients’ needs. The MOH always ensures that healthcare services delivery, including COVID-19 treatment provided, is at its optimal.

### KN2 Discoveries and Technological Advances in Medicine: The Way Forward to Cater for Pandemics

#### Asma Ismail^1,2^ (asmainformm@yahoo.com)

##### ^1^Ibnu Sina Professorial Chair in Medicine, International Islamic University Malaysia, 25200 Kuantan, Pahang, Malaysia; ^2^Academy of Sciences Malaysia, Level 20, MATRADE Tower, Jalan Sultan Haji Ahmad Shah, 50480 Kuala Lumpur, Malaysia

**Abstract**

Pandemics are events of global public health and economic importance with 75% due to zoonotic in origin but exacerbated due to human actions. Despite having many lessons learned from the history of pandemics, prevention of future pandemics needs collective societal responses. The ability to tackle issues and challenges in attaining collective societal response will be the tipping point for preventable measures of future pandemics. Unless the concerns raised take heed we need to have an alternate plan and investigate the use of modern technologies to fight against future pandemics. With the advent of industry 4.0, disruptive technologies have entered into our daily lives and livelihoods with numerous successes. In short, modern technologies and discoveries have been used to identify, track and forecast outbreaks. It is used in helping diagnosis of the virus, deliver food and medicine supplies, sterilizing public places and even in processing the healthcare claims. Drug discovery and vaccine developments have been accelerated using super computers. There is indeed a wide scope of potential utilizations of technologies to combat future pandemics even though many are still in its infancy and could not show its full operational effect today. Despite the availability of current technological advancements there are still issues and challenges in the country that need to be handled as measures of pandemic preparedness in order to show resilience.

## Plenaries

### P1 Collaboration between University and Government Agencies During Pandemic

#### Hanafiah Harunarashid (drhanafiah@yahoo.com)

##### Pro Vice-Chancellor’s Office, Universiti Kebangsaan Malaysia Kuala Lumpur Campus, 56000 Cheras, Kuala Lumpur, Malaysia

**Abstract**

Why is the issue of collaboration raised? Firstly, there is no doubt that rigid bureaucratic boundaries do exist, and perhaps enhanced during crisis. University hospitals have played an important role in the fight against COVID-19 but are barely mentioned as part of frontline asset. University hospitals typically function as district hospitals with tertiary care set up. They are public funded, stand-alone set ups and provide subsidized care. The pandemic has enhanced its ties with the primary network. Using UKM as an example, this lecture will highlight how we managed to collaborate with the Ministries of Health and Higher Education during the earlier part of the crisis. We also had close links with the Non-Governmental organisations, local authorities, MOSTI, pharmaceutical companies, individual philanthropists and of course with our sister teaching hospitals via the KHUAM network. HCTM is a hybrid hospital managing COVID 19 cases including some high-profile clusters and UKM laboratories had contributed in reducing the RT PCR testing backlog at the government laboratories. Good communication and coordination are the key to a successful outcome. It may be unfathomable for the rest of the world that Malaysia can perform well when other established countries with greatest resources and wealth falter in the face of this global peril of the new millennium. Whether we fare better or worse in global ranking, the fact remains that the fate and wellbeing of the *rakyat* is being threatened by unseen forces, not only by autonomous ravaging virus particles but also a global economy at the brink of collapse. How we fared as a country so far is a question of national pride. There is much to do, and much to learn to forge ahead with our fragile future as a country. The prayers to save Malaysia in the past have been answered, and with grace of the Almighty may we given the strength and wisdom to overcome the many challenges ahead from outside and within.

### P2 COVID-19 Vaccine as A Solution for Returning to the Old Normal

#### Norazmi Mohd Noor (norazmimn@usm.my)

##### School of Health Sciences, Universiti Sains Malaysia Health Campus, 16150 Kota Bharu, Kelantan, Malaysia

**Abstract**

The roll out of COVID-19 vaccines is eagerly awaited by many countries to allow life to return to what we regard as the “old” normal. Encouraging data is beginning to emerge in the first few countries which are aggressively vaccinating their population. At the time of writing, Israel, for example, has vaccinated almost 90% of their population, and has observed reduction in the number of severe cases among vaccinees 70 years and older. Other encouraging observations are also being collected. All these initial reports augur well for the vaccination campaign. However, there are still a few unknowns. What is the duration of the protection following immunisation? Would vaccination with the current types of vaccines protect against the new virus variants? What are the correlates of protection for COVID-19 to allow for proper monitoring of vaccinees? These unanswered questions remain the main reasons why return to the “old” normal may not be as soon as we hope for.

### P3 Update on COVID-19 Management

#### Adeeba Kamarulzaman (adeeba@ummc.edu.my)

##### Infectious Diseases Unit, Universiti Malaya Medical Centre (UMMC), 59100 Lembah Pantai, Kuala Lumpur, Malaysia

**Abstract**

The COVID-19 pandemic has tested the medical fraternity like no other disease in recent years. Although the large majority of patients who become infected suffer from mild or even no symptoms, approximately 20 percent of patients may develop severe complications including respiratory failure, thrombo-embolic diseases and multi-organ failure. Although viral in origin, the clinical manifestation of COVID-19 is multi-system in nature. Understanding the patho-physiology and natural history of the disease is therefore crucial in order to provide the best care by a multi-disciplinary team. The presentation will describe the natural history and clinical manifestations of COVID-19 infection and provide an update on treatment and management of patients with severe disease.

### P4 Big Data and Precision Medicine: Leveraging Opportunities

#### A. Rahman A. Jamal (rahmanj@ppukm.ukm.edu.my)

##### UKM Medical Molecular Biology Institute (UMBI), Universiti Kebangsaan Malaysia, Bandar Tun Razak, 56000 Cheras, Kuala Lumpur, Malaysia

**Abstract**

If there are two key drivers that will transform healthcare, Big Data and Precision Medicine will arguably be top of the list. As teaching hospitals, we are familiar with the Big Data available in our hands whether these are still paper-based or already in the digital format. Millions of datasets and terabytes of data are available from the hundreds of thousands of patients that come to the hospital every year. To start with, there is the opportunity to digitize patients’ data through the implementation of the electronic medical record (EMR) and the spectrum of modules required under the total hospital information system (THIS). Transforming unstructured data into structured data and then digitizing the data will be a challenge but can be done. Once these data (clinical, laboratory, radiological, pharmacy, demography, outcome, etc.) are available, the opportunities for big data analytics are huge and will attract researchers from many disciplines including those from the social sciences. The precision medicine initiative deals directly on how we should transform patient care to make it personalized, predictive, preventive and participatory. The aim of precision medicine is to administer the right treatment to the right individual at the right time, using the unique genetic, environmental exposure and lifestyle of each of them. This will optimize the outcome and survival, minimize adverse reactions, allow targeted therapies for conditions such as cancers, provide a predictive risk score to each individual hence allowing them to exercise preventive approaches. During the pandemic COVID-19, the opportunities for big data analytics and application of precision medicine are staring at us. As of 23 February 2021, more than 112 million cases have been confirmed, with more than 2.48 million deaths attributed to COVID-19. As teaching hospitals, we should play the leadership roles and leverage on these opportunities hence obtain the best outcomes.

## Symposium 1

### S1S1 Primary Care Practitioner Life During COVID-19 Outbreak: Preparation and Challenges

#### Nur Suhaila Idris (nursuhaila@usm.my)

##### Family Medicine Department, Hospital Universiti Sains Malaysia, Universiti Sains Malaysia Health Campus,16150 Kota Bharu, Kelantan, Malaysia

**Abstract**

The unprecedented crisis of the COVID-19 Pandemic has posed a challenge to the healthcare systems worldwide including the primary health care services. As the first point of contact, the primary care services under the Department of Family Medicine acted fast in order to prevent the COVID-19 infection transmission by proactively identifying potential COVID-19 cases whilst ensuring the essential health services are not disrupted. At Hospital USM, in order to achieve the aim, multiple strategies like restructuring and coordinating the health services have been implemented including creating a primary triage at the entrance of outpatient clinics, QR-coded risk declaration form for patients and visitors, online risk declaration form and COVID-19 Hotline for the staff and students. Other measures are separating the infectious and non-infectious cases via the newly set up *Klinik Saringan Infeksi(SRI)* or Infection Screening Clinic with the innovation applied and relocation of the blood taking service outside of the clinic. The new norm i.e physical distancing at the waiting area and teleconsultation for stable chronic diseases follow up are also created. Like other primary health care facilities, to aid the MOH COVID-19 screening, the sampling activities and surveillance for the staff and students with the COVID-19 related risk are also provided as a main task, also for income generation for the hospital and university from the sampling activities done under Corporate unit and USAINS. The primary care team are also equipped and kept updated with the latest guidelines, SOP’s, sampling training and online CME’s. We also continued our community health education activities via social media to ensure an optimal and holistic task are achieved. The uncertainty of COVID-19, high risk exposure (screening activities, fake risk declaration of patients) and other emerging issues make the struggle a real challenge; physically, mentally and emotionally for the primary care team. However, with the great multidisciplinary teamwork and support from the higher authority, we can win this battle together.

### S1S2 The Challenges of COVID-19 Hospitals and Other Healthcare Facilities in Facing the Pandemic

#### Mahiran Mustafa^1,2^ (mahiranmustafa@hotmail.com)

##### ^1^Service in Infectious Disease, Ministry of Health, 62200 Putrajaya, Malaysia; ^2^Internal Medicine Department, Hospital Raja Perempuan Zainab II, 15586 Kota Bharu, Kelantan, Malaysia

**Abstract**

The COVID-19 pandemic is just 15 months old. The pandemic has demonstrated, resilient and sustainable health systems have never been more important. It takes stakeholders to understand what measures must be implemented in order to minimize the disruption. Predicting numbers of new cases and Rt /Ro enable the health facilities to be prepared. Ensuring the supply and demand of laboratory test, infrastructure, beds, manpower, equipment and PPE that match each other are exhaustive exercise. Universal infection prevention and control practice has gone from health facility to the community. Having multiple hospital acquired outbreak of COVID-19 in MOH facility make our number of healthcare workers (HCW) much less and thus lead to fatigue and burnout among HCW. The evolution and new variants of SARs COV-2 make us on alert mood to look at transmission precaution, treatment guidelines and choice of vaccine. Sadly, in the absence of consistent, all-of-society necessities, the trip will be longer and costly. Players from multi-agencies should show more solidarity in supporting and collaborate in fighting the pandemic. The health systems must reform to be more prepared for next pandemic.

### S1S3 COVID-19 Implications on Health Tourism

#### Ismail Sagap^1,2^ (drisagap@yahoo.com)

##### ^1^Department of Surgery, Faculty of Medicine, University Kebangsaan Malaysia (UKM) Kuala Lumpur, Jalan Yaacob Latif, Bandar Tun Razak, 56000 Cheras, Kuala Lumpur, Malaysia; ^2^Medical Director of UKM Specialist Center, Hospital Canselor Tuanku Muhriz (HCTM), National University of Malaysia Medical Centre, 56000 Cheras, Kuala Lumpur, Malaysia

**Abstract**

In 2019, Malaysia Healthtourism achieved more than MYR1.7 billion in hospital receipts, resulting total economic impact of MYR7 billion. Unprecedented turn of events such as the closing of international borders have disrupted several of the nation’s key economic sectors including healthcare travel, which saw an impact of over 50% in revenue reduction. However, it has played to its strengths, ensuring industry resilience and future recovery through digital services and platforms to address the needs of healthcare travellers. For 2021, even with vaccine availability, a lot of this will depend on the policies in place to safeguard both Malaysia and the home countries of our medical travellers, and of course, the coverage that the vaccine provides, both from an efficacy perspective, and also how widespread the vaccines are applied. It is predicted and hoped that the numbers return to at least RM1 billion by 2022.

### S1S4 Collaborative Effort in Instilling Vaccine Confidence – The Fight Against Vaccine Misinformation

#### Zulkifli Ismail^1,2^ (drzulkifli.ismail@gmail.com)

##### ^1^Malaysian Paediatric Association, Menara Arina Uniti 97, Jalan Raja Muda Abdul Aziz, 50300 Kuala Lumpur, Malaysia; ^2^KPJ Selangor Specialist Hospital, Seksyen 20, 40300 Shah Alam, Selangor, Malaysia

**Abstract**

The World Health Organisation (WHO) recognized that vaccine hesitancy is one of ten threats to global health in 2019. A key element in the rise of vaccine hesitancy and refusal (VHR) is the widespread misinformation and disinformation using social media as an efficient dissemination tool. This has created a widening trust deficit on vaccination resulting in a decrease in vaccine confidence. Increasing vaccine confidence requires a collaborative effort, recognizing the strengths of individuals and organizations, and capitalizing on these. An effective vaccine engagement and advocacy program requires ample professional knowledge on vaccinology combined with credibility and authority of the members and adequate funding. One such organization has been active in Malaysia since June 2013, called Immunise4Life (www.ifl.my). It is a tripartite collaboration involving the authority of the Ministry of Health Malaysia, content provided and scrutinized by the Malaysian Paediatric Association (MPA) and the Malaysian Society of Infectious Diseases & Chemotherapy (MSIDC), and financial support from most industry stakeholders, the whole thing being supported by an efficient Health Communications secretariat. The involvement of other social media influencers working together providing similar messaging and amplifying each other’s messages will result in a huge ripple effect to counter disinformation from the opposite direction. Vaccine messaging to counter anti-vaccine influence needs to use the same medium to be effective, i.e. social media. In addition, there is still place for traditional media to ensure that no group is left out. Factual and staid messaging will lose out to story-telling format when dealing with the public. Even audiovisual media have superseded written paragraphs when dealing with the younger generation. There is a need for more active rebuttals to anti-vaccine messaging so as to reduce VHR and maintain our high vaccination rates to ensure adequate herd immunity, and the eventual eradication and elimination of vaccine-preventable diseases.

## Symposium 2

### S2S1 Socio-Economic Impacts of COVID-19 on Household Consumption and Poverty

#### Rashidah Shuib (rashidahhshuib@gmail.com)

##### Interdisciplinary Health Sciences Unit, School of Health Sciences, Universiti Sains Malaysia Health Campus, 16150 Kota Bharu, Kelantan, Malaysia

**Abstract**

In 2020, the coronavirus COVID-19 rapidly and steadily spread, stretching into 2021, resulting in a global pandemic that has changed the world and people’s lives. The real impact on the economy and people’s lives, however, is yet to be fully understood as the situations are layered in complexities and uncertainties; continuously evolving. The length of “stay-at-home” policy, the types of business allowed to operate, and the duration of economic recovery are amongst the multitude of factors driving the uncertainties, making it difficult to predict the impact on individual households. What is known for sure is that millions of people are suffering globally. In Malaysia and in other parts of the world, jobs are lost, workers are retrenched or have their pay being cut drastically, businesses are closed, borders are shut and controlled making staying at home and social distancing the new norm. COVID-19 has shown deep societal crevices that divide the society, existing inequalities and inequities deepened, impacting hard on the vulnerable groups and the poor. The pandemic impacts the disadvantaged communities and different income groups differently. Using the existing limited literature in Malaysia, this paper attempts to explore how and in what ways COVID-19 has impacted household consumption and poverty given Malaysia’s socio-economic-political scenario prior to COVID-19, the context of government’s stimulus measures and the differential impact of the pandemic. The paper postulates that the university hospitals have critical roles to play in helping those vulnerable households and the poor, those malnourished and the increase in mental health cases.

### S2S2 Sustainable Higher Education: Teaching and Learning During and Post COVID-19 Pandemic

#### Muhamad Saiful Bahri Yusoff (msaiful_bahri@usm.my)

##### Department of Medical Education, School of Medical Sciences, Universiti Sains Malaysia Health Campus, 16150 Kota Bharu, Kelantan, Malaysia

**Abstract**

The new norm, due to the recent pandemic, is inevitable and has changed the way people live. It leads to social distancing, personalized space, small group-based activities, and increase the use of digital platforms in daily activity. It has also given a great impact on the way higher education being carried out around the globe. During the first wave of COVID-19, many universities made compulsory the online teaching and learning but for a lot of lecturers, they had difficulty adapting to it. Whilst for the students (undergraduates and postgraduates), the online methods reduce the learning outcomes especially those related to skill-based applications and acquisition of new skills. The challenges and impacts on higher education sustainability during and after the pandemic are highlighted and, the alternatives and the way forward are discussed.

### S2S3 COVID-19 Behavioural Insights and Residential Aged Care Situation: A Preliminary Analysis

#### Rahimah Ibrahim (imahibrahim@upm.edu.my)

##### Malaysian Research Institute of Ageing (MyAgeing^TM^), Universiti Putra Malaysia, 43400 Serdang, Selangor Darul Ehsan, Malaysia

**Abstract**

Although there are more COVID-19 positive cases among the younger age groups (20-39), the majority of deaths (84.95%) occurred in the population aged 50 above. The death rate is very much higher in the older age groups from 8.4% (aged 60-69) to 21.37% (aged 70-79), to 46.26% (aged 80+). Given the vulnerability of the aged population towards COVID-19, the primary purpose of the research is to improve the early recognition, prevention, response, and control of COVID-19 in the community and among older persons living in institutions. Between the 15^th^ of December 2020 until 1^st^ of February 2021, 412 respondents completed the national survey covering experiences*, current practices, and perceptions towards COVID-19 and the public health measures initiated by the government. Out of the 412 respondents, the majority were female, aged 22-39, from the Malay ethnic group, and had tertiary education. Preliminary analysis of the data reveals that age, gender, education level, and household classification have a bearing on the perception of infection susceptibility and severity, media intensification of COVID-19, compliance with Movement Control Order (MCO) rulings, decision to vaccinate, and sentiments toward rulings or sanctions. Despite the lack of data on outbreaks, public records and internal discussion groups identified at least 30 known outbreaks around the country that have happened in residential aged care facilities. These outbreaks put a strain of resources on the aged care sector and vaccination of aged care staff and residents is seen as crucial in reducing the number of recorded deaths in the country. Therefore, public health measures to improve public awareness and sentiments towards COVID-19 rulings and sanctions, as well as the decision to vaccinate, should take into account socio-demographic factors and the higher death rate amongst older age groups.

### S2S4 Planning for What Comes After COVID-19

#### Tunku Kamarul Zaman Tunku Zainol Abidin^1,2^ (tkzrea@um.edu.my)

##### ^1^Secretariat of Public Universities’ Hospital Consortium (Konsortium Hospital Universiti Awam Malaysia, KHUAM), Universiti Malaya Medical Centre (UMMC), 59100 Lembah Pantai, Kuala Lumpur, Malaysia; ^2^Department of Orthopaedic Surgery, Faculty of Medicine, Universiti Malaya, 59100 Lembah Pantai, Kuala Lumpur, Malaysia

**Abstract**

Globally, people’s everyday life has been overturned by the pandemic COVID-19. In the midst of pandemic chaos, there is much for us to cogitate and contemplate. While the pandemic is indeed a wake-up call for us to immediately reassess our behavior with the environment and adopt new way of life, developing self-discipline in resisting temptations is very challenging. We are obliged to continue to support lives and ensuring that people continue to receive healthcare that they deserve in the aftermath of this unforeseen global conundrum. Therefore, how can we carefully reposition ourselves to ensure we can continue to work unhindered or continue to believe in the power of planning? In a period of uncertainty, it is impossible to predict the future. Nevertheless, it is important to evaluate the range of possibilities in order to be ready for what comes after COVID-19. The aim is not to be able to play all the scenarios but to become more agile and to adapt to a new scenario with alacrity, even one which has not been anticipated. This disease has altered and even devastated many facets of human civilization; may it be from the standpoint of public health, welfare, infrastructure development, technological progress and even economics. As public teaching university hospitals are concerned, after the pandemic has passed, hospitals can expect that hordes of patients will throng their entrances demanding that healthcare centres deal with their health problems. All of which have been put on hold and have become complicated due to various circumstances. Many will come to these institutions with financial hardships and turning to hospitals for assistance. With such a poor economic environment, we can all expect to manage our hospitals with lesser resources but with higher demands from the *rakyat*. In addition, services will need to be delivered in accordance with new norms. These add to existing burden of present operational needs and that to the cost of healthcare delivery. All of these add up to the complexities of the present problem and run the risk of institutional collapse. At the end of this global vaccination program, of which in Malaysia is expected to complete by March 2022, we are hoping to see an end to this pandemic. Nonetheless, we can also expect the rippling effect of the aftershock to follow the devastation left behind. The question is; Are we prepared for this? In a period of uncertainty, not doing anything is a decision worse than making the wrong decision and thus, being prepared is ultimately the only choice that all of us have. In this presentation, we shall explore the possible scenarios that can occur after COVID-19 and what we can do now to be prepared for it.

## Symposium 3

### S3S1 Establishing Laboratory Support for COVID-19: From Zero to Hero?

#### Azian Harun (azian@usm.my)

##### Medical Microbiology & Parasitology Department, School of Medical Sciences, Universiti Sains Malaysia Health Campus, 16150 Kota Bharu, Kelantan, Malaysia

**Abstract**

The COVID-19 outbreak has quickly spread worldwide since its discovery in Wuhan City, China in December 2019. Malaysia was not spared, and the health authorities were in need to rapidly implement diagnostic tools. Diagnostic laboratories in Malaysia have been in various states of readiness to respond to the mass testing challenge. Major steps in establishing the COVID-19 molecular testing laboratory include identifying a suitable space, necessary renovation, staff training, procurement of equipment and materials. This presentation offers a look inside a laboratory responding to COVID-19 pandemic, with sharing of experience, challenges and prospects in the rapid establishment of COVID-19 testing laboratory.

### S3S2 Laboratory Preparedness for Potential Pandemic Agents

#### Sazaly Abu Bakar (sazaly@um.edu.my)

##### Tropical Infectious Diseases Research & Education Centre (TIDREC), High Impact Research (HIR) Building, Universiti Malaya, 50603 Kuala Lumpur, Malaysia

**Abstract**

In 2005, the World Health Organization (WHO) through all its member states adopted the legally binding legal framework, the International Health Regulation (2005). All member states are required to have the ability to 1): Ensure surveillance systems can detect acute public health events in a timely manner (Detect), 2) Assess public health events and report to WHO of events that may constitute a public health emergency of international concern (Assess and report), and 3) Respond to public health risks and emergencies (Respond). In compliance with the IHR requirements, Malaysia established the Malaysia Strategy for Emerging Diseases (MySED) I Workplan (2012-2015) and the more recent MySED II (2017- 2021) in tandem with the implementation of the WHO WPRO Asia Pacific Strategy for Emerging Diseases and Public Health Emergencies (APSED). The COVID-19 pandemic which started in earnest in early 2020 offered a real-time opportunity to assess the utility of the MySED Workplan for Malaysia. Among the core components of the workplan is laboratory preparedness. Here we highlighted and described the relevance and importance of university laboratories in serving as the next line of responder in the face of a raging pandemic. These laboratories are usually reference laboratories that can perform select agent research andable to complement pandemic screening programs performed by the public health laboratories. These experiences brought to light the need to include these laboratories in the overall strategy and workplan for future pandemic plan preparedness beyond MySED II.

### S3S3 Impact of Bioinformatics in the COVID-19 Pandemic and Future Epidemic Responses

#### Mohd Firdaus Mohd Raih^1,2^ (m.firdaus@ukm.edu.my)

##### ^1^Institute of Systems Biology, Universiti Kebangsaan Malaysia, 43600 Bangi, Selangor, Malaysia; ^2^ Centre for Frontier Sciences, Faculty of Science and Technology, Universiti Kebangsaan Malaysia, 43600 Bangi, Selangor, Malaysia

**Abstract**

The COVID-19 pandemic has seen an unprecedented volume and rate of data deposition in the relevant major public repositories. Bioinformatics has played a key role in translating the data into actionable strategies and deployable applications. The diverse contributions of bioinformatics in studying this disease includes but are not limited to vaccine design, genomic tracking, mutant surveillance, functional characterization, drug design and drug repurposing. In this seminar, the impact of these contributions are reviewed in a global context, how they are applicable in a national setting and what the future holds for the bioinformatics in the management and response to future epidemics.

## Symposium 4

### S4S1 Public Health Academics in the COVID-19 War

#### Awang Bulgiba Awang Mahmud (awang@um.edu.my)

##### Department of Social and Preventive Medicine, Faculty of Medicine, Universiti Malaya, 50603 Kuala Lumpur, Malaysia

**Abstract**

The first COVID-19 wave in Malaysia started on 25 January 2020 and died down within a month. The second wave forced Malaysia into its first ever Movement Control Order (MCO) which successfully controlled the outbreak. However, the third wave which began in September 2020 has proved to be a real challenge. In response to the pandemic, a whole lot of public health academics have come together to help the country. These public health academics have helped with epidemiological analysis and strategies especially during the second wave and have taken on the job of surveilling health care workers in their own teaching hospital from the second wave onwards. At the same time, public health academics have been at the forefront in attempting to influence government policies and educating the public. When vaccination became a reality, public health academics were involved in proposing vaccination policies and plans for the government. Some of the policy advocacy has been accomplished via two taskforces set up by the Ministry of Science, Technology and Innovation (MOSTI) – the CEASe (COVID-19 Epidemiological Analysis & Strategies) Taskforce and ICVAC (Independent COVID-19 Vaccination Advisory Committee). Other policy advocacy work has involved the media as a partner. The COVID-19 pandemic has forced the country to confront the tremendous tasks that lie ahead as it tries to recover the ground and the time that was lost during the pandemic. Malaysia needs to maintain a strong health system, socially responsible population and good governance in order to achieve the right balance between public health action, economic survival and social equilibrium.

### S4S2 Traumatic Stress in Healthcare Workers During COVID-19 Pandemic and Support Mechanism

#### Asrenee Ab Razak^1^, Syaheedatul Iman Dinsuhaimi^1^, Azhani Yaakub^2^, Azizah Othman^3^, Aziah Daud^4^, Kamarul Imran Musa^4^, Wan Zahiruddin Wan Mohammad^4^, Nani Deraman^5^, Alwi Besari^6^, Liza Sharmini Tajudin^2^

##### ^1^Department of Psychiatry; ^2^ Department of Opthalmology; ^3^ Department of Paediatric; ^4^ Department of Community Medicine; ^5^Department of Family Medicine; ^6^Department of Medicine, School of Medical Sciences, Kota Bharu 16150 Kelantan, Malaysia

###### **Correspondence:** Asrenee Ab Razak (asrenee@usm.my)

**Abstract**

In COVID-19 pandemic, undoubtedly, healthcare workers (HCW) are among those who are exposed to infection risk. Hence the psychological reaction is not uncommon especially related to fear of infection which escalate high anxiety, traumatic stress and depression. In addition, long working hours in uncomfortable environment, misunderstanding or wrong information and perceived lack of support exaggerate the condition. Thus, it is a need to explore the psychological impacts of COVID-19 pandemic among HCW in our local setting. A cross sectional study was conducted in Hospital USM in April 2020 via an online survey. Self-rated Depression, Anxiety and Stress Scale-21 (DASS 21) and Malay Impact of Event Scale Revised (M-IESR) were used to assess the psychological distress. A total of 818 HCW responded to the survey, with the mean age of 35.45 (SD: 8.15) year-old. Majority are from clinical based (91.4%) and (21.3%) are exposed to patients with COVID-19 risk. Majority reported mild or moderate distress with Movement Control Order, mild degree of traumatic stress with IESR mean score of 22.73 (SD: 16.88) and low mean score of depression, anxiety and stress with 2.70 (SD: 3.87), 2.74 (SD: 3.58) and 3.33 (SD: 3.98) respectively. Out of 818, only 46 found to be depressed, 80 anxious and 15 stressed. Previous history of mental illness and dealing with patients with COVID 19 risk have significant association with depression and anxiety. Whilst being single is associated with depression only and younger age is associated with anxiety only. It is worth to note that the data collection was done while Hospital USM was functioning as non-COVID-19 hospital. The findings however indicate the risk of developing psychological distress among HCW is associated with previous history of mental illness and being exposed to COVID-19 risk patients. Therefore it is crucial to support their psychological wellbeing especially after the massive surge of cases in the second and third waves. Among the measures employed in the hospital are the activation of psychological first aid team, proactive approach with tele-counselling, hotline services, and educational webinar series. In conclusion, the HCW are vulnerable to develop psychological distress during COVID-19 pandemic especially those who deal with COVID-19 patients and with history of mental illness. The proactive measures of psychological first aid should be considered as early as possible in order to support their wellbeing and to make sure the health service is uninterrupted.

### S4S3 Humanizing Public Education in the Times of Crisis: The Role of Social Media

#### Mohd Fauzi Bin Kamarudin (mohdfauzi@utem.edu.my)

##### Centre for Language Learning (CeLL), Universiti Teknikal Malaysia Melaka (UTeM), 76100 Durian Tunggal, Melaka, Malaysia

**Abstract**

Education worldwide is embracing rapid change due to globalization, competition, market orientation and technology. Education in the modern world puts emphasis on maximizing learners’ potential through immersive learning. Recently, in the pandemic world of COVID 19 and its impact towards education, there have been greater calls to humanise public education to ensure that education continues to be personalised, contextualised, dynamic and geared towards a personalized experience for students. Nevertheless, managing the betterment of education is not an easy task since public education needs to constantly engage with various stakeholders. Additionally, social media has been called upon to play an important role towards the effort of humanising education. This paper presents an overview of the findings in current research focusing on the role of social media towards humanising public education. The paper concludes by suggesting several factors important for social media practitioners in relation to their role as influencers and research tool in the world of pandemic crisis.

## Forum

### F1 Responsibilities, Challenges and Future Directions of Academic Teaching Hospital During Pandemic

#### Kamarul Aryffin Baharuddin^1,2^, Ahmad Sukari Halim^1,2^, Nazirah Hasnan^3^, Razman Jarmin^4^, Mohamed Saufi Awang^5^

##### ^1^School of Medical Sciences, Universiti Sains Malaysia, Health Campus, Kubang Kerian, Kelantan, Malaysia; ^2^ Hospital Universiti Sains Malaysia, Health Campus, Kubang Kerian, Kelantan; ^3^ Universiti Malaya Medical Centre, Lembah Pantai, 59100 Kuala Lumpur; ^4^ Hospital Canselor Tuanku Muhriz UKM, Jalan Yaacob Latif, Bandar Tun Razak, 56000 Kuala Lumpur, Malaysia; ^5^ Sultan Ahmad Shah Medical Center @ IIUM, International Islamic University Malaysia, Jalan Sultan Ahmad Shah, 25200 Kuantan, Pahang, Malaysia

###### **Correspondence:** Kamarul Aryffin Baharuddin (amararyff@usm.my)

**Abstract**

This forum discussed about the challenges faced by teaching hospitals in Malaysia during COVID-19 pandemic. University Malaya Medical Centre (UMMC) was the first non-MOH hospital recognized as COVID-19 referral centre in January 2020. In response, UMMC had to reorganize its clinical services started with zoning of the hospital blocks to dedicate COVID-19 and non-COVID-19 zones followed by identifying dedicated team. Other measures for reorganization were (1) redeployment, training and cohorting of the staff (2) rescheduling of clinics and reduction in admission (3) cancellation of elective surgeries (4) gazettement of the COVID-19 wards and (5) engineering control. Subsequently, on March 20^th^, 2020, Hospital Canselor Tuanku Muhriz became the second non-MOH hospital for COVID-19 referral centre, followed by the Sultan Ahmad Shah (SAS) Medical Centre@IIUM on March 22nd, 2020. However, the risk of infection among the healthcare providers at these two teaching hospitals was significant. Sultan Ahmad Shah (SAS) Medical Centre@IIUM was the first to badly affected which led to shut down of services including teaching activities. Aggressive contact tracing and disinfection activities were carried out and within a short period of time, the hospital resumed as usual. Hospital Canselor Tuanku Muhriz was significantly affected during the 3rd wave of the pandemic. To overcome the situation, the hospital applied the TERAS strategy – Talent, Ethics, Revitalize, Agile, Soul. At Hospital USM, the director discussed about the hospital’s governance during the COVID-19 pandemic. Overall, for these hospitals, operational matters were significantly affected as the budget allocation and human resource allocation of teaching hospitals were much less compared to MOH hospitals. Policy and action plan are dependent on MOH and autonomous among each other’s. Decision of patient transfer are bound to inter agency negotiation. In the event of surge of cases, there was limited capacity to divert to the other facility due to stand-alone entity.

